# Disseminated Cryptococcal Disease in a Patient with Chronic Lymphocytic Leukemia on Ibrutinib

**DOI:** 10.1155/2016/4642831

**Published:** 2016-09-14

**Authors:** Koh Okamoto, Laurie A. Proia, Patricia L. Demarais

**Affiliations:** ^1^Division of Infectious Diseases, John H. Stroger Jr. Hospital of Cook County, Chicago, IL, USA; ^2^Division of Infectious Diseases, Rush University Medical Center, Chicago, IL, USA

## Abstract

*Cryptococcus* is a unique environmental fungus that can cause disease most often in immunocompromised individuals with defective cell-mediated immunity. Chronic lymphocytic leukemia (CLL) is not known to be a risk factor for cryptococcal disease although cases have been described mainly in patients treated with agents that suppress cell-mediated immunity. Ibrutinib is a new biologic agent used for treatment of CLL, mantle cell lymphoma, and Waldenstrom's macroglobulinemia. It acts by inhibiting Bruton's tyrosine kinase, a kinase downstream of the B-cell receptor critical for B-cell survival and proliferation. Ibrutinib use has not been associated previously with cryptococcal disease. However, recent evidence suggested that treatments aimed at blocking the function of Bruton's tyrosine kinase could pose a higher risk for cryptococcal infection in a mice model. Here, we report the first case of disseminated cryptococcal disease in a patient with CLL treated with ibrutinib. When evaluating possible infection in CLL patients receiving ibrutinib, cryptococcal disease, which could be life threatening if overlooked, could be considered.

## 1. Introduction

*Cryptococcus* is a unique environmental fungus that can cause disease of any organ system, with the lungs and central nervous system (CNS) most commonly affected [1, 2]. Among species of* Cryptococcus*,* C. neoformans* accounts for the majority of human infections.* C. gattii *has had more attention in the last decade due to an outbreak that began in the west coast of North America in 1999. Although* Cryptococcus* can cause disease in immunocompetent individuals, especially* C. gattii*,* C. neoformans* infection occurs most often in immunocompromised individuals, primarily those with defective cell-mediated immunity such as human immunodeficiency virus (HIV) infection [1, 2]. Other populations known to be at risk for cryptococcal disease include solid organ and stem cell transplant recipients, patients receiving immunosuppressive agents, and patients with advanced malignancies [3, 4]. Here, we report a case of disseminated cryptococcal disease in a patient with chronic lymphocytic leukemia (CLL) on ibrutinib.

## 2. Case Report

A 68-year-old African American woman with well controlled type II diabetes mellitus, hypertension, and hyperlipidemia was diagnosed with CLL (Rai stage III with negative CD38 and ZAP-70) in September 2012 when she presented with night sweats and 4.5 kg weight loss over the preceding eight months. She received six cycles of chlorambucil and prednisone over the following two years with partial response in her white blood cell count (WBC). Polymorphonuclear cells (PMN) and lymphocytes ranged from 1.1 to 4.7 × 10^3^/*μ*L and 32 to 125 × 10^3^/*μ*L, respectively. In October 2014, she was started on ibrutinib 420 mg daily. In late November 2014, she was admitted for fever of 39.6°C and sore throat. After admission, she developed septic shock which was thought to be due to hospital acquired pneumonia and presumed* Clostridium difficile* colitis. Endotracheal culture grew* Klebsiella pneumoniae*, methicillin susceptible* Staphylococcus aureus*, and* Candida krusei*. She required mechanical ventilation, hemodynamic support with norepinephrine and vasopressin, chest tube placement, and broad spectrum antibiotics (vancomycin and piperacillin/tazobactam). She recovered slowly and was discharged to a nursing home after two weeks of hospitalization. Ibrutinib was held during this hospitalization while PMN and lymphocytes ranged from 2.2 to 4.7 × 10^3^/*μ*L and 9.6 to 24.1 × 10^3^/*μ*L. One week after discharge, in December 2014, WBC increased to 67 × 10^3^/*μ*L; hence, ibrutinib was restarted. In January 2015, she presented with dry cough for one month and lower lip numbness for one hour. In the emergency department, she had no focal neurological deficit and CT brain without contrast showed no acute intracranial pathology; however, she had fever of 39.1°C, tachycardia, and tachypnea and was admitted. Blood pressure and oxygen saturation on ambient air were normal. Physical examination revealed expiratory wheezing in the right lower lung field; WBC on admission was 20.4 × 10^3^/*μ*L with 24% of neutrophil and 65% of lymphocyte. CT chest without contrast showed consolidation within the superior segment of the left lower lobe suggestive of pneumonia. She was started on empiric vancomycin and piperacillin/tazobactam and later transitioned to ceftazidime for presumed healthcare associated pneumonia, with gradual improvement of fever. Given patient's clinical response to empiric antibiotics, no further diagnostic testing for pneumonia was performed. However after five days, one set of admission blood cultures grew yeast and oral fluconazole (800 mg load and then 400 mg daily) was initiated on the same day. She was later discharged to a nursing home on oral levofloxacin, clindamycin, and fluconazole pending identification of the yeast. Subsequently, the yeast blood isolate was identified as* Cryptococcus neoformans*. She was readmitted and underwent lumbar puncture after 8 days of fluconazole. Cerebral spinal fluid (CSF) analysis was unremarkable with WBC of 1/*μ*L, opening pressure was normal, and CSF and serum cryptococcal antigen were negative. Repeat blood cultures and CSF fungal culture were negative. Immunoglobulin G and M levels were low (immunoglobulin G level 558 mg/dL, reference range 594–1,618 mg/dL; immunoglobulin M level 47 mg/dL, reference range 77–220 mg/dL) but immunoglobulin A level was within normal range (333 mg/dL, reference range 68–378 mg/dL). CD4 lymphocyte count was not obtained. She received a 2-week course of induction therapy with liposomal amphotericin (3 mg/kg/day) and flucytosine for disseminated cryptococcal disease and was discharged on oral fluconazole 400 mg daily. Ibrutinib was restarted at lower dose given potential hepatic cytochrome P450 interaction with fluconazole. She remained clinically stable at one-year follow-up visit.

## 3. Discussion

CLL can cause defects in both cell-mediated and humoral immunity [5]. More than 30 cases of cryptococcal disease in patients with CLL have been described [6, 7]. While some patients had never been treated for CLL prior to diagnosis of cryptococcal disease, most cases occurred in patients who were treated extensively with various immunosuppressive agents [8]. In particular, purine analogues, such as fludarabine, or alemtuzumab, which mainly affect cell-mediated immunity, pose higher risk [9]. In contrast, invasive fungal infections appear to be uncommon in patients who receive conventional alkylator therapy, such as chlorambucil [9]. Ibrutinib is a newer biologic agent used to treat CLL, mantle cell lymphoma, and Waldenstrom's macroglobulinemia. Ibrutinib inhibits Bruton's tyrosine kinase, a kinase downstream of the B-cell receptor critical for B-cell survival and proliferation, which is thus important for humoral immunity [10]. In recently published clinical trials, the most common infectious complication was pneumonia occurring in 6–12% of patients with CLL treated with ibrutinib [11, 12]. There are no reports to date of invasive fungal infection occurring in patients treated with this agent. To our knowledge this is the first report of disseminated cryptococcal disease in a CLL patient treated with ibrutinib.

Cell-mediated immunity plays a central role in preventing and controlling infection caused by* Cryptococcus* [1, 2]. Interestingly, there is growing evidence indicating a role for humoral immunity as well [13, 14]. Most recently, Szymczak et al. examined the importance of B-1 B-cells for resistance to* C. neoformans* infection by using X-linked immunodeficient mice carrying a mutation in Bruton's tyrosine kinase [15]. They found that immunodeficient mice were unable to contain* Cryptococcus *in the lungs with reduced uptake by macrophages, progressive lung infection, and dissemination to the brain. They concluded that treatments aimed at blocking the function of Bruton's tyrosine kinase could pose a higher risk for cryptococcal infection. Although rare, a case of cryptococcal empyema in a child with Bruton's agammaglobulinaemia has been reported in the literature [16].

Our patient had disseminated cryptococcosis; it is likely that she had cryptococcal pneumonia with secondary fungemia. We hypothesize that treatment of this patient's CLL with ibrutinib might have increased susceptibility to* Cryptococcus* given the evidence of association between Bruton's tyrosine kinase function and onset of cryptococcal disease while CLL itself and low level of immunoglobulin could have been contributing factors. Our case also suggests that inhibition of Bruton's tyrosine kinase with widespread use of ibrutinib might lead to increased risk for other invasive fungal infections in this patient population. Guidelines for the prevention and treatment of cancer-related infections from the National Comprehensive Cancer Network recommend consideration of fungal prophylaxis only during neutropenia and for anticipated mucositis in patients with CLL [17]. Hence, most patients with CLL are unlikely to be on antifungal prophylaxis. In conclusion, cryptococcal disease is uncommon among patients with CLL; however, our case suggests possible increased susceptibility to this disease with ibrutinib. Cryptococcal disease, which could be life threatening if overlooked, could be considered in such patients.
